# Supplementary material for: The role of explicit memory in syntactic persistence: Effects of lexical cueing and load on sentence memory and sentence production
Source: PLoS One. 2020 Nov 5;15(11):e0240909. doi: 10.1371/journal.pone.0240909 (PMC7643978; doi:10.1371/journal.pone.0240909)
Supplement: S1 Appendix — (DOCX) [file pone.0240909.s001.docx]

**S1 Appendix.** **Summary of fixed effects in LME models in the subset analyses of Experiment 1a-b and 2a-b.**

|  | Estimate | SE | z | p-value |
| --- | --- | --- | --- | --- |
| *Experiment 1a – Difficult problem* |  |  |  |  |
| (Intercept) | -4.977 | 0.711 | -7.005 | <.001 |
| Prime | 5.919 | 1.203 | 4.919 | <.001 |
| Head noun condition | -0.063 | 1.051 | -0.060 | .952 |
| Processing time | 0.854 | 0.363 | 2.350 | .019 |
| Prime: Head noun condition | 3.453 | 2.077 | 1.662 | .096 |
| Prime: Processing time | -1.922 | 0.705 | -2.728 | .006 |
| Head noun condition: Processing time | 0.213 | 0.658 | 0.323 | .746 |
| Prime: Head noun condition: Processing time | -0.017 | 1.315 | -0.013 | .990 |
|  |  |  |  |  |
| *Experiment 1b – Difficult problem* |  |  |  |  |
| (Intercept) | -1.600 | 0.371 | -4.316 | <.001 |
| To-be-recalled structure | 5.647 | 0.654 | 8.641 | <.001 |
| Head noun condition | 1.162 | 0.476 | 2.443 | .015 |
| Processing time | -0.116 | 0.233 | -0.499 | .618 |
| To-be-recalled structure: Head noun condition | 1.374 | 0.885 | 1.553 | .120 |
| To-be-recalled structure: Processing time | 0.084 | 0.449 | 0.186 | .852 |
| Head noun condition: Processing time | -0.619 | 0.429 | -1.445 | .148 |
| To-be-recalled structure: Head noun condition: Processing time | 1.327 | 0.835 | 1.589 | .112 |
|  |  |  |  |  |
| *Experiment 2b – Same Head Noun* |  |  |  |  |
| (Intercept) | -1.355 | 0.414 | -3.273 | .001 |
| Prime | 8.965 | 0.791 | 11.328 | <.001 |
| Difficulty | -0.140 | 0.388 | -0.361 | .718 |
| Prime: Difficulty | 1.901 | 0.777 | 2.445 | .014 |
|  |  |  |  |  |
| *Experiment 2a – Different Head Noun* |  |  |  |  |
| (Intercept) | -2.021 | 0.293 | -6.895 | <.001 |
| Prime | 5.798 | 0.544 | 10.660 | <.001 |
| Difficulty | 0.032 | 0.277 | 0.115 | .909 |
| Prime: Difficulty | 0.201 | 0.544 | 0.370 | .712 |
