# Supplementary material for: The role of explicit memory in syntactic persistence: Effects of lexical cueing and load on sentence memory and sentence production
Source: PLoS One. 2020 Nov 5;15(11):e0240909. doi: 10.1371/journal.pone.0240909 (PMC7643978; doi:10.1371/journal.pone.0240909)
Supplement: S2 Appendix — The description of the target picture is given in first line depicts the content of the target picture. The possessor of the colored object and the object that is owned are mentioned. In the following lines, the s-genitive (a) and the of-genitive primes (b) are given in Dutch. In each prime sentence, the noun in the Same Head Noun condition is mentioned before the slash and the noun in the Different Head Noun condition is mentioned after the slash. (DOCX) [file pone.0240909.s002.docx]

**S2 Appendix. Primes and targets used in each experiment**

The description of the target picture is given in first line depicts the content of the target picture. The possessor of the colored object and the object that is owned are mentioned. In the following lines, the s-genitive (a) and the of-genitive primes (b) are given in Dutch. In each prime sentence, the noun in the Same Head Noun condition is mentioned before the slash and the noun in the Different Head Noun condition is mentioned after the slash.

1. Girl with a red pineapple

1a. De zuster haar ananas/paraplu is rood.

1b. De ananas/de paraplu van de zuster is rood.

2. Wizard with a blue apple

2a. Het meisje haar appel/ijsje is blauw.

2b. De appel/het ijsje van het meisje is blauw.

3. Wizard with a red beard

3a. De jongen zijn baard/mais is rood.

3b. De baard/de mais van de jongen is rood.

4. Nurse with a yellow banjo

4a. De piraat zijn banjo/spiegel is geel.

4b. De banjo/de spiegel van de piraat is geel.

5. Pirate with a green barbecue

5a. De heks haar barbecue/wasknijper is groen.

5b. De barbecue/de wasknijper van de heks is groen.

6. Witch with a blue bear

6a. De non haar beer/kwast is blauw.

6b. De beer/de kwast van de non is blauw.

7. Girl with a red bomb

7a. De tovenaar zijn bom/taart is rood.

7b. De bom/de taart van de tovenaar is rood.

8. Boy with a blue lemon

8a. De non haar citroen/ketel is blauw.

8b. De citroen/de ketel van de non is blauw.

9. Witch with a red duck

9a. De jongen zijn eend/kaas is rood.

9b. De eend/de kaas van de jongen is rood.

10. Witch with a yellow egg

10a. De zuster haar ei/paard is geel.

10b. Het ei/het paard van de zuster is geel.

11. Nurse with a blue bucket

11a. Het meisje haar emmer/konijn is blauw.

11b. De emmer/het konijn van het meisje is blauw.

12. Pirate with a red giraffe

12a. De priester zijn giraf/kussen is rood.

12b. De giraf/het kussen van de priester is rood.

13. Wizard with a green guitar

13a. De jongen zijn gitaar/ezel is groen.

13b. De gitaar/de ezel van de jongen is groen.

14. Boy with a blue glass

14a. De heks haar glas/bijl is blauw.

14b. Het glas/de bijl van de heks is blauw.

15. Priest with a yellow hand

15a. De tovenaar zijn hand/doos is geel.

15b. De hand/de doos van de tovenaar is geel.

16. Girl with a blue heart

16a. De non haar hart/mier is blauw.

16b. Het hart/de mier van de non is blauw.

17. Boy with a blue shirt

17a. De piraat zijn hemd/duim is blauw.

17b. Het hemd/de duim van de piraat is blauw.

18. Nun with a green yo-yo

18a. De piraat zijn jojo/handdoek is groen.

18b. De jojo/de handdoek van de piraat is groen.

19. Nun with a blue gift

19a. De tovenaar zijn cadeau/kleerkast is blauw.

19b. Het cadeau/de kleerkast van de tovenaar is blauw.

20. Pirate with a yellow church

20a. De zuster haar kerk/sok is geel.

20b. De kerk/de sok van de zuster is geel.

21. Pirate with a red cage

21a. De non haar kooi/hond is rood.

21b. De kooi/de hond van de non is rood.

22. Girl with a green ladder

22a. De priester zijn ladder/sneeuwpop is groen.

22b. De ladder/de sneeuwpop van de priester is groen.

23. Witch with a green knife

23a. De zuster haar mes/tand is groen.

23b. Het mes/de tand van de zuster is groen.

24. Wizard with a red nest

24a. De zuster haar nest/bot is rood.

24b. Het nest/het bot van de zuster is rood.

25. Witch with a green hippopotamus

25a. De priester zijn nijlpaard/ketting is groen.

25b. Het nijlpaard/de ketting van de priester is groen.

26. Witch with a green eye

26a. Het meisje haar oog/slak is groen.

26b. Het oog/de slak van het meisje is groen.

27. Nun with a green palm tree

27a. De priester zijn palmboom/tafel is groen.

27b. De palmboom/de tafel van de priester is groen.

28. Pirate with a yellow pan

28a. De heks haar pan/jas is geel.

28b. De pan/de jas van de heks is geel.

29. Nurse with a yellow paintbrush

29a. De heks haar penseel/kalkoen is geel.

29b. De penseel/de kalkoen van de heks is geel.

30. Boy with a red pipe

30a. De priester zijn pijp/slot is rood.

30b. De pijp/het slot van de priester is rood.

31. Boy with a red band aid

31a. Het meisje haar pleister/potlood is rood.

31b. De pleister/het potlood van het meisje is rood.

32. Boy with a blue pop

32a. De tovenaar zijn pop/touw is blauw.

32b. De pop/het touw van de tovenaar is blauw.

33. Nurse with a green belt

33a. De jongen zijn riem/steen is groen.

33b. De riem/de steen van de jongen is groen.

34. Nurse with a red robot

34a. De tovenaar zijn robot/koffer is rood.

34b. De robot/de koffer van de tovenaar is rood.

35. Priest with a blue rose

35a. De jongen zijn roos/haak is blauw.

35b. De roos/de haak van de jongen is blauw.

36. Priest with a yellow sheep

36a. Het meisje haar schaap/stoel is geel.

36b. Het schaap/de stoel van het meisje is geel.

37. Priest with a blue turtle

37a. De heks haar schildpad/vliegtuig is blauw.

37b. De schilpad/het vliegtuig van de heks is blauw.

38. Witch with a red shoe

38a. De zuster haar schoen/hert is rood.

38b. De schoen/het hert van de zuster is rood.

39. Priest with a yellow scarf

39a. De jongen zijn sjaal/zaag is geel.

39b. De sjaal/de zaag van de jongen is geel.

40. Pirate with a blue skate

40a. De non haar skatebord/papfles is blauw.

40b. Het skatebord/de papfles van de non is blauw.

41. Pirate with a yellow snake

41a. De non haar slang/traan is geel.

41b. De slang/de traan van de non is geel.

42. Boy with a yellow skipping rope

42a. De priester zijn springtouw/beker is geel.

42b. Het springtouw/de beker van de priester is geel.

43. Boy with a yellow thermos

43a. Het meisje haar thermos/wortel is geel.

43b. De thermos/de wortel van het meisje is geel.

44. Girl with a red fly

44a. De non haar vlieg/zweep is rood.

44b. De vlieg/de zweep van de non is rood.

45. Girl with a green fork

45a. De zuster haar vork/boom is groen.

45b. De vork/de boom van de zuster is groen.

46. Nurse with a green bag

46a. Het meisje haar zak/schroef is groen.

46b. De zak/de schroef van het meisje is groen.

47. Nun with a yellow flashlight

47a. De piraat zijn zaklamp/kogel is geel.

47b. De zaklamp/de kogel van de piraat is geel.

48. Pirate with a green zebra

48a. De tovenaar zijn zebra/vuilbak is groen.

48b. De zebra/de vuilbak van de tovenaar is groen
