# Supplementary material for: The role of explicit memory in syntactic persistence: Effects of lexical cueing and load on sentence memory and sentence production
Source: PLoS One. 2020 Nov 5;15(11):e0240909. doi: 10.1371/journal.pone.0240909 (PMC7643978; doi:10.1371/journal.pone.0240909)
Supplement: S3 Appendix — The appendix only displays each problem in an addend order that the first addend is always larger than the second. In the reported experiment the order of the addends was counterbalanced. (DOCX) [file pone.0240909.s003.docx]

**S3 Appendix. Arithmetic problems used in each experiment**

The appendix only displays each problem in an addend order that the first addend is always larger than the second. In the reported experiment the order of the addends was counterbalanced

|  | Experiment 1a, 1b | |  |  | Experiment 2a, 2b | |  |
| --- | --- | --- | --- | --- | --- | --- | --- |
| Easy | | Difficult | | Easy | | Difficult | |
| 11 + 1 | | 36 + 11 | | 11 + 1 | | 39 + 13 | |
| 11 + 2 | | 54 + 11 | | 11 + 2 | | 69 + 13 | |
| 12 + 1 | | 71 + 12 | | 12 + 1 | | 79 + 14 | |
| 12 + 2 | | 62 + 12 | | 12 + 2 | | 28 + 14 | |
| 13 + 1 | | 55 + 13 | | 13 + 1 | | 39 + 15 | |
| 13 + 2 | | 74 + 13 | | 13 + 2 | | 48 + 15 | |
| 14 + 1 | | 62 + 14 | | 14 + 1 | | 27 + 15 | |
| 14 + 2 | | 85 + 14 | | 14 + 2 | | 28 + 15 | |
| 16 + 1 | | 31 + 16 | | 15 + 1 | | 17 + 16 | |
| 16 + 2 | | 53 + 16 | | 15 + 2 | | 25 + 16 | |
| 17 + 1 | | 61 + 17 | | 16 + 1 | | 39 + 16 | |
| 17 + 2 | | 32 + 17 | | 16 + 2 | | 77 + 16 | |
| 21 + 1 | | 44 + 21 | | 17 + 1 | | 56 + 17 | |
| 21 + 2 | | 36 + 21 | | 17 + 2 | | 26 + 17 | |
| 22 + 1 | | 65 + 22 | | 18 + 1 | | 54 + 18 | |
| 22 + 2 | | 46 + 22 | | 21 + 1 | | 79 + 18 | |
| 23 + 1 | | 74 + 23 | | 21 + 2 | | 59 + 23 | |
| 23 + 2 | | 34 + 23 | | 22 + 1 | | 39 + 23 | |
| 25 + 1 | | 31 + 25 | | 22 + 2 | | 48 + 24 | |
| 25 + 2 | | 34 + 25 | | 23 + 1 | | 27 + 24 | |
| 26 + 1 | | 41 + 26 | | 23 + 2 | | 48 + 25 | |
| 26 + 2 | | 71 + 26 | | 24 + 1 | | 56 + 25 | |
| 27 + 1 | | 61 + 27 | | 24 + 2 | | 47 + 25 | |
| 27 + 2 | | 42 + 27 | | 25 + 1 | | 67 + 25 | |
| 31 + 1 | | 65 + 31 | | 25 + 2 | | 37 + 26 | |
| 31 + 2 | | 37 + 31 | | 26 + 1 | | 57 + 26 | |
| 33 + 1 | | 36 + 33 | | 26 + 2 | | 27 + 26 | |
| 33 + 2 | | 46 + 33 | | 27 + 1 | | 59 + 26 | |
| 34 + 1 | | 65 + 34 | | 27 + 2 | | 35 + 27 | |
| 34 + 2 | | 52 + 34 | | 28 + 1 | | 65 + 27 | |
| 35 + 1 | | 43 + 35 | | 31 + 1 | | 29 + 28 | |
| 35 + 2 | | 54 + 35 | | 31 + 2 | | 33 + 28 | |
| 36 + 1 | | 61 + 36 | | 32 + 1 | | 59 + 33 | |
| 36 + 2 | | 51 + 36 | | 32 + 2 | | 39 + 33 | |
| 37 + 1 | | 42 + 37 | | 33 + 1 | | 58 + 34 | |

**S3 Appendix (continued)**

|  | Experiment 1a, 1b | |  |  | Experiment 2a, 2b | |  |
| --- | --- | --- | --- | --- | --- | --- | --- |
| Easy | | Difficult | | Easy | | Difficult | |
| 37 + 2 | | 62 + 37 | | 33 + 2 | | 48 + 34 | |
| 41 + 1 | | 57 + 41 | | 34 + 1 | | 38 + 35 | |
| 41 + 2 | | 47 + 41 | | 34 + 2 | | 39 + 35 | |
| 43 + 1 | | 46 + 43 | | 35 + 1 | | 49 + 35 | |
| 43 + 2 | | 44 + 43 | | 35 + 2 | | 57 + 35 | |
| 44 + 1 | | 53 + 44 | | 36 + 1 | | 49 + 36 | |
| 44 + 2 | | 45 + 44 | | 36 + 2 | | 37 + 36 | |
| 45 + 1 | | 54 + 45 | | 37 + 1 | | 45 + 36 | |
| 45 + 2 | | 53 + 45 | | 37 + 2 | | 58 + 36 | |
| 46 + 1 | | 53 + 46 | | 38 + 1 | | 56 + 37 | |
| 46 + 2 | | 52 + 46 | | 41 + 1 | | 46 + 37 | |
| 47 + 1 | | 51 + 47 | | 41 + 2 | | 44 + 38 | |
| 47 + 2 | | 52 + 47 | | 42 + 1 | | 55 + 38 | |
