# Supplementary material for: The role of explicit memory in syntactic persistence: Effects of lexical cueing and load on sentence memory and sentence production
Source: PLoS One. 2020 Nov 5;15(11):e0240909. doi: 10.1371/journal.pone.0240909 (PMC7643978; doi:10.1371/journal.pone.0240909)
Supplement: Attachment — Submitted filename: Response to reviewers _Zhang et al..docx [file pone.0240909.s004.docx]

Reviewer #1: This paper investigates the contribution of explicit memory to structural priming. The authors describe four experiments in which they investigate the relationship between structural priming, lexical boost, and the difficulty of a concurrent memory load. In two experiments (one dialogue, one monologue), participants performed a standard structural priming task, describing pictures using one of two genitive alternations. In the other two experiments (again one dialogue, one monologue), participants were explicitly told to repeat the sentence structure of the preceding sentence. In all experiments, in between the prime and target sentence, participants solved an addition problem, which was easy or hard; the difference in difficulty between the two types of math problems was increased for the third and fourth experiment.
A robust structural priming and lexical boost effect was found. The authors also found some evidence of task difficulty – i.e., degree of cognitive load – interacting with degree of syntactic priming and lexical boost, to the effect that subjects were less likely to repeat the prime structure when they cognitive load was more difficult, either because they spent longer engaged in the cognitive load task (exploratory RT analyses) or it was a more difficult math problem.
This is an interesting and well-written paper which addresses a theoretically relevant question about the mechanisms underlying structural priming and its reliance on explicit memory. The research questions are well-grounded in prior research and the experiments seem well-designed. The discussion and interpretation is well-reasoned from the results and interesting as well. However, I think there are issues with the statistics which call into question the main result – that higher cognitive load causes reduced structural priming/recall – and thus I do not think much can be inferred from the results and concomitant conclusions of this paper. I believe a complete re-analysis of the statistics is required, and that may (in my opinion, likely will) completely change the central conclusion of the paper.

Major comments:
#1 Table 1: This table seems to show that in the Same Head Noun condition, 36.0% of the subjects’ productions were s-genitive and 4.7% were of-genitive, and 15.7% and 5.5%, respectively in the Different Head Noun condition.
(a) Why are these values so low? That means that subjects were producing non-s/of-genitive structures on 60-80% of the trials (depending on condition), correct? What were these other structures?
(b) I can’t reconcile these data with the earlier statements that only 8.6% of responses were “other” and that 84.4% of responses were of-genitive (it looks like from Table 1 that ~5% in each condition were of-genitive).
(c) If subjects are producing a different structure on such a large proportion of the trials, please give some assurance that their behavior is indicative of a larger priming processing effect given how many trials were UNaffected by the prime manipulation.

<<Author’s response: These comments stem from a misinterpretation of Table 1, presumably due to our unclear title of the table and labeling of the columns. The Table lists the proportion of s-genitives out of all s-genitives and of-genitives, for each experiment, level of lexical overlap condition (rows), and level of prime condition (columns). Thus, the value of 36% the reviewer is referring to means that in the Same Head Noun, s-genitive prime condition, 36% of valid responses were s-genitives (and so 64% were of-genitives). But in the Same Head Noun, of-genitive prime condition, only 4.7% of the responses were s-genitives (and so 95.3% were of-genitives). Thus, there was a priming effect of about 30%. This of course also means that the number of others was really 8.6% - the proportions listed in the Table are based only on non-others (s-genitives and of-genitives). We have revised the title and the header of the table. We hope this clarifies the table.>>

#2 The authors write (pg. 20, line 473-476): “we included in the model the by-subject and by-item random intercepts as well as random slopes for all main effects and interactions in the fixed model. The full model (and all the other full models in this paper) converged.” But when I look at the code posted on OSF (thank you!), this does not seem to be the case. There are a ton of models there, but it looks like the full model that is being used in the model comparison ANOVAs is DP0122, which has all random slopes removed (i.e., everything except the intercept). It has the full set of fixed effects, but the random effects are just:
(1 | Subject) + (1 | Item)
Similarly, for all of the sub-models that were run, with the fixed effect in question removed, also have the random effects structure of just (1 | Subject) + (1 | Item).
(a) The authors shouldn’t write that all random slopes were included, as it appears that was not the case for the actual model comparisons. It should be clear as to the model structure that was used for the statistics that are reported.
(b) If the models that included all the random slopes converged, then why is that model not being used for the statistics? Removing random slopes can make a model output anti-conservative and may skew the results.
(c) Note that this point is relevant to all of the experiments, as the code that was posted said it was used for all four experiments.

<<Author’s response: We thank the reviewer for pointing this out. In fact, the script in the OSF data repository contained an older version of our analysis, in which we employed backward selection for the models. In the version of the analyses that we reported in the manuscript we did employ the maximal random model suggested by Barr et al. (2013). We are very sorry that we did not update the OSF files in time. We have now put the correct R scripts in the OSF repository, one for each pair of experiments. In the script we included all the necessary codes for the descriptive and inferential analyses in the manuscript.>>

#3 Likely related to the previous point, I find the results for Experiment 2a to be rather surprising, given the figures and data. I’m not sure what the error bars represent, but assuming they’re standard error, I find it quite unlikely that those would produce an interaction between Prime Condition x Difficulty or a main effect of Difficulty, and yet those are robustly significant.

<<Author's response: The error bars denote the standard of the error mean from a by-participants analysis; this has now been added to the Figure caption. In interpreting the figure, please keep in mind that the Y-axis represents the priming effect. Thus, the interaction between prime condition and difficulty is reflected in a difference between the easy and difficult conditions. The main effect of difficulty (which is of no theoretical interest) cannot be gauged from the figure.>>

When I run the code using the model with no random slopes (DP0122), there is indeed a significant Prime Condition x Difficulty interaction. However, I could not get the full model to converge when I ran it myself (the maximal model with all random slopes + intercepts which the text said had converged). So to try to approximate that model, I ran an F1 ANOVA, which, given that there was a balanced design and minimal data loss (2.2%), and I wouldn’t expect big item differences in your stimuli, the ANOVA should show pretty similar results to the LMER. Some results were very similar, as expected (Prime Condition, Prime Condition x Head Noun Condition) but all of the effects involving Task Difficulty were extremely different, e.g. the main effect of Task Difficulty in the ANOVA was F < 1, p = .92; Prime Condition x Task Difficulty was F = 2.5, p = .12; Prime Condition x Head Noun Condition x Task Difficulty was F < 1, p = .75, and these results seem much more in line with the descriptive data. I am not suggesting that the authors run ANOVAs instead of LMERs; there are many good reasons to run LMERs for this type of data. However, it’s important to make sure the results make sense given what the raw data show. As is noted in the next paragraph: “Unexpectedly, we also found a significant main effect of task difficulty (χ2 = 4.966, df = 1, p = .025) and a significant two-way interaction between task difficulty and lexical overlap (χ2 = 7.213, df = 1, p = .007). There was no clear theoretical reason for these effects, and similar effects were not found in any other experiments. We decided to refrain from speculation about these unexpected findings, which might well be a result of type I error.” Given that Task Difficulty shows a tiny effect in the numerical data, but yet shows a significant (or marginal) result for all of the fixed effects that it's involved in, plus none of them were significant in the sample ANOVA that I ran, and you have the minimal random effects structure in your models, and you even note that some of the Task Difficulty effects were unexpected and might be type I error – I think there is something wrong in the LMERs as conducted. It just doesn’t pass the eyeball test. As I cannot get the maximal models to converge on my machine, I can’t see what result that returns myself, so the advice I have is two-fold:
(a) look into the analyses and really make sure they’re being done correctly and that the statistical results match common-sense intuition based on the numerical data
(b) run the model-comparison stats on the maximal model that was reported to have converged, and see if the effects still hold as currently reported.

<<Author’s response: We appreciate it that the reviewer went to the trouble of checking the analyses and even conducted an ANOVA as an approximation. We apologize again that the scripts on OSF were outdated. We have checked all analyses, and observed that our statistical inferences regarding task difficulty do hold - although these reanalyses did bring up a new issue, namely that of singular fit, which led to a number of changes to the inferential statistics, and for one interaction a difference in significance (see below). We respond in more detail below:

(a) Model convergence

In the data analyses employed in the manuscript we kept the maximal random model for each linear mixed model. We accepted the model results that showed the warning *singular fit* since the model converged. But experts on linear mixed models have raised caution about the singular fit in model convergence. Some argued it might indicate that a complicated model is overfitted and might result in mis-convergence (Bates et al., 2015). Thus, in the current version of the data analyses, we followed Barr et al. (2013) by keeping the most complex random model that did not result in singular fit. To do this, we started with the maximal random model and simplified the random model only when the model failed to converge or the model generated a singular fit. If the model showed singularity, we first dropped the random correlations then dropped one random factor a time, starting from the most complex interaction term, until the non-singular model was fitted. We updated the modelling method as well as the structure and fixed effects of the final models in the manuscript and appendices.

(b) The three-way interaction in Experiment 2a

The only place where there might be a discrepancy between the observed data and the model estimation was in the three-way interaction between prime condition, head noun condition, and problem difficulty in Experiment 2a. Descriptively, the difficult problem reduced the numerical size of the structural priming effect to a similar extent in the Same Head Noun condition and in the Different Head Noun condition. However, in the latest data analysis, the three-way interaction became significant in the model comparison analysis (i.e., model comparison between the reduced model and the full model). A possible interpretation of the three-way interaction is that the two-way interaction between prime condition and problem difficulty differed in the subsets divided by head noun condition. Although we did not make a strong argument out of this significant three-way interaction, we nevertheless fitted two additional models that predicted the likelihood of s-genitive production in the head noun condition subsets. We found that the interaction between the prime condition and problem difficulty was only significant in the Same Head Noun subset. The results of the subset models were added in the manuscript, and the fixed effect of the models were put in Appendix A. >>

#4 pg. 38, line 924-925 says: “The non-significant interaction between cognitive load and lexical overlap on structural priming was somewhat surprising…” and then discusses the implications of this non-effect. I assume that is referring to the interaction between Task Difficulty x Head Noun Condition in Experiment 2a. However, when reporting the Exp 2a results, the text says (line 740): “Unexpectedly, we also found … a significant two-way interaction between task difficulty and lexical overlap (χ2 = 7.213, df = 1, p = .007).” Please resolve this conflict or clarify which comparison you are referring to in the GD.

<<Author’s response: For the first interaction the reviewer refers to what we meant was rather the three-way interaction between prime condition, head noun condition, and problem difficulty. Sorry we did not make it clear. Now we have changed the expression to “It is somewhat surprising that the cognitive load effect on structural priming was not predominantly head-specific.” Thanks for pointing this out.>>

Minor comments:
#5 pg. 25, line 593: The exploratory analysis of Exp 1b investigating processing time (I assume also by using RT, as in Exp 1a) x to-be-recalled structure has df = 0.42. I do not understand how doing a model-comparison test can produce a non-integer degrees of freedom. (Unfortunately I can’t look at the model output to help me understand as I can’t find it in the submission.)

<<Author’s response: This was a typo. We have revised this result.>>

-There are a few times throughout the paper when results between experiments are compared numerically, but not statistically (e.g., pg. 26, comparing the size of the lexical boost effect in Exps 1a vs 1b, etc.). Please make those comparisons statistically explicit and report the significance/non-significance of the cross-experiment comparison.

<<Author’s response: Based on this comment, we have added two sections of cross-experiment analyses (Page 25-26 and Page 35) that compares the structural priming effects, lexical boost effects, and cognitive load effects between the structural priming experiments and sentence structure memory experiments. In both analyses, there were significant two-way interaction between prime condition and experiment, but the three-way interactions between prime condition, head noun condition, and experiment were not significant. We also calculated the Bayesian factors of these interactions to further examine to what extent these effects supported the alternative hypotheses.>>

#6 I wonder how difficult even the most difficult math problems were. Is carrying in addition really such a challenging task? Subjects are only spending 3 seconds doing the math problem in the hardest case. Please talk a little about why you think this might be enough of a cognitive load to disrupt processing.

<<Author’s response: In the design of the experiments we needed to find a balance between having an effortful task and having a relatively short task, given the transiency of the lexical boost effects. We needed to keep the difficult secondary task relatively short but effortful. The processing of a carrying problem is around 1900 ms slower than solving an easy problem. This suggests that a considerable amount of cognitive resource is taxed in solving carrying problems. Additionally, a similar manipulation of cognitive load significantly affected the preparation time of sentence production (Ferreira & Swets, 2002), we believe it is possible that the cognitive load manipulation in the current study makes a difference on the choice of syntactic structures.>>

#7 In only Exp 2b is there the expected context effect, whereby production of the less-common structure increased over the course of the experiment; in the other experiments, this effect goes in the opposite direction (the less-common structure decreases over the experiment). This is surprising, given that it’s the opposite direction as would be expected. The authors write that the increase effect (the expected direction) is “possibly indicating a practice effect.” Please discuss this further. Why would there be a practice effect in Exp 2b but not the other experiments with (approximately) the same task? Why would the other experiments show the opposite direction as previously-attested context effects?

<<Author’s response: The reversed cumulative effect on the production of the less preferred sentence structure (i.e., s-genitive) might be attributed to a surprisal effect. When the participants first perceived an s-genitive prime, they would be more prompted to use this structure in the ensuing task because the unexpected first encounter exerts a strong prediction error. As the experiment progressed, the participants might then regress to their preferred structure to alleviate the processing load in sentence production, resulting in a decreasing pattern of s-genitive production.

We argue such a reversed cumulative effect would not occur in the sentence structure memory experiments. Instead, a practice effect might manifest itself in these experiments. The production tasks in the sentence structure memory experiments was driven by an additional goal to accurately repeat the previously encoded sentence structure. We would expect that the accuracy of structure repetition gradually increased as the participants were getting familiarized with the task, resulting in higher chance to produce the less frequent structure as the experiment progressed. We added a brief discussion about these effects at Page 37 (for the practice effect) and Page 44 (for the reversed cumulative effects). >>

Textual/editing comments:
#8 The references are not all standardized – some use the journal abbreviation (J Mem Lang) and others the full name (Journal of experimental psychology: Learning, memory, and cognition).

<<Author’s response: We have changed all journal names into the abbreviated form as required by the journal's submission format.>>

-pg. 14: references example stimuli sentences before the sentences are produced in the text. This makes that entire paragraph difficult to follow; I suggest moving the sample sentences before the paragraph describing the stimuli conditions (i.e., at line 330).

<<Author’s response: Fixed. Thank you for pointing that out.>>

#9 pg. 17-18, lines 419-427: There’s a strange paragraph, maybe it’s supposed to be the figure caption for Figure 2? It’s presented in the middle of the text (with no accompanying figure, which is only at the end of the text body).

<<Author’s response: Yes, it is the figure caption for Figure 2; the journal's submission guidelines require us to put them in the text immediately after the paragraph in which the figure was first cited>>

#10 pg. 21, line 501 (same for Exp 1b): The authors write that the model for the supplementary analysis investigating RT of Exp 1a is in the Supplementary Material, but it’s not in Appendix A, B, or C (which are the only attached materials), so please include an explicit pointer to where I can find these results.

<<Author’s response: We apologize for this omission. We have now added these models in Appendix A.>>

#11 Please note in the figure captions what the error bars are: standard error, standard deviation, confidence interval, etc.

<<Author’s response: We have noted in the figure caption that the error bars reflect standard errors calculated for a by-participants analysis.>>

#12 Please make the y-axis for the two figures on the same scale. It makes it easier to compare the results across experiments.

<<Author’s response: Fixed. Thank you for pointing that out.>>

#13 Grammar/wording comments:
-pg. 5, line 102-3: “It has been several decades for the debate about how such structural priming effect comes about.” -> “It has been several decades since the debate began about…” (for example)
-pg. 7, line 164: “tried to answer A similar question”
-pg. 9, line 224: “These findings are coherent with…” consistent with?
-pg. 13, line 310, 318: “figurines” -> figures
-pg. 27, line 651: “we predicted that the secondary task posited a cognitive load” -> imposed a cognitive load?
-pg. 30, line 726: “minuses” -> minus
-pg. 35, line 856: “more often correctly” -> delete “correctly” perhaps?
-pg. 37, line 881: intertwinement -> entwining
-pg. 38, line 915-956: “similar as for the recall of sentence structure” -> “similar TO” ?
-pg. 39, line 932: “whereas the memory of sentence structure is prune to the load manipulation.” -> I am not sure what this sentence intends.

<<Author’s response: We have modified all the grammar errors.>>

Reviewer #2: • I appreciate the availability of the authors’ data.
#1 Overall, I agree with the authors that it is interesting to further explore cognitive load factors that affect priming. I believe the manuscript presents a nice beginning to this. I am, however, not convinced by Experiment 1a in this manuscript. The authors employ a confederate, which I appreciate. I think it is prudent to consider social factors. Perhaps some participants were high self-monitors and simply took more time answering questions to not be judged poorly by their experimental partner. If this were a factor, it may be that they were paying less attention to their partner’s descriptions in anticipation of their turns.

<<Author’s response: This is an interesting point. We argue below however that even in the absence of a physical interlocutor, the participants' utterances still had communicative value. Additionally, a reanlysis does not find evidence for differences in secondary task difficulty. Specifically, in Experiment 2a and 2b the participants were instructed that the utterances they would listen to came from a “participant” in the earlier test (who was in fact a confederate) and that their recording would be used in the upcoming tests of further participants. So we would argue that the production tasks in Experiment 2a and 2b also carried communicative purpose. The main difference in the social contexts of Experiments 1a-b and 2a-b would be the physical presence of an interlocutor. To examine whether the appearance of an interlocutor influenced participants’ processing time in problem solving, we conducted a between-experiment comparison of the processing time in filler arithmetic problem solving tasks. The materials in these filler problem solving tasks were identical in all four experiments. This enabled us to conduct a pairwise comparison of by-item mean processing time between experiments. We only examined the processing time of the hard problems (with or without carrying) because solving an easy problem requires little attention resources. If the participants took more time answering the questions when collaborating with an interlocutor, the processing time of a hard problem in the dialogue experiments should be longer than that in the monologue experiments. To test this hypothesis, we aggregated the processing time across items and compared the mean processing time between Experiment 1a and 2a as well as between 1b and 2b. The mean processing time in Experiment 1a was 3095 ms and that in Experiment 2a was 3097 ms. The standardized difference was negligible (Cohen's d for paired samples = -0.006). The difference of processing time between Experiment 1b (3102 ms) and 2b (3103 ms) was also negligible (Cohen's d for paired samples = -0.002). This indicates that the participants in the dialogue experiments did not take more time than those in the monologue experiments to solve the same filler task, suggesting no effect of the presence of an interlocutor on secondary task processing.>>

• Regarding the second series of experiments – I found it interesting that the authors decided to make two major changes – 1) the difficulty of the problems and 2) the dismissal of the confederate. I agree that the arithmetic problems, especially the “easy” ones, likely presented minimal, if any cognitive load on the participants. However, making two changes at once leads to questionable conclusions – how can you be sure which manipulation led to the change in significance?

<<Author’s response: We have argued in the response to comment #1 that the physical presence of an interlocutor had little impact on secondary task processing, and hence made little difference for the change of the cognitive load in Experiment 2a-b compared to Experiments 1a-b. We believe the main factor that drove the load increase was the more difficult secondary task.>>

• I think this is a useful addition to the literature. However, I would recommend running a third study with a confederate and the more difficult problems. It would round off these series of experiments nicely and address the concerns I (and the authors even noted) have about the manuscript.

<<Author’s response: Thank you for pointing this out. But because of the limited effect of this manipulation on the problem solving task, we have decided not to follow this suggestion>>
