# Supplementary material for: The role of explicit memory in syntactic persistence: Effects of lexical cueing and load on sentence memory and sentence production
Source: PLoS One. 2020 Nov 5;15(11):e0240909. doi: 10.1371/journal.pone.0240909 (PMC7643978; doi:10.1371/journal.pone.0240909)
Supplement: Attachment — Submitted filename: Response to Reviewers for Second Revision _Zhang et al..docx [file pone.0240909.s005.docx]

Reviewer #1: This paper is still an interesting topic and contribution to the literature. However, there remain several inconsistencies or errors with the analyses which have the potential to affect the results. I feel the authors need to fix the analyses before I can have confidence that the results are in fact as they have been presented (and thus of course whether the discussion/interpretation follows).

MAJOR COMMENTS:
#1 There still is confusion about the running and reporting of the lmer models. Due to the discrepancies between what is reported in the paper for the overall analysis strategy, what is reported for each individual experiment, and the R code, in addition to the fact that the reported results often seem at odds with the means and sds shown in the tables/figures (as even the authors themselves note at times), I believe the authors need to very carefully check their analyses, and this must be resolved before I can have confidence in them. I detail several (potentially related) concerns below.

The authors write (pg. 22, line 112) "If the maximal model could not converge or showed singularity, we first dropped the random correlation terms, and then dropped one random factor at a time, starting from the interaction terms, until the model converged and no warning of singular fit was reported." They write something similar in their Response letter.

a) How did you decide which order to drop terms if a model did not converge? A common strategy is to iteratively drop the random effect which accounts for the least amount of variance, but it does not sound like this was what was done. The authors say their strategy was to start by dropping the most complex interaction term, but how did you decide the order of dropping terms at the same complexity level (i.e., the order of dropping among the 3 2-way interactions, or among the 3 main effects)? It looks like terms were dropped based on the order that they were written in the model starting from the bottom-up; in order words, arbitrarily within a complexity level. That is not a good strategy, as dropping random terms can affect significance and thus should not be done arbitrarily. Dropping a random effect which accounts for a substantial amount of variance can have a big effect on the observed significance of fixed effects, and while this is necessary sometimes for convergence/singularity reasons, it needs to be done systematically.

<<Authors’ response: We did adopt a bottom-up strategy of term dropping in the random model in the previous model selections. The reason we employed this method was to avoid the potential complication when a lower-level random effect accounts for less variance than a higher-level random effect.

In the newest version of the manuscript, we adapted the method to your suggested strategy. Now, in the LME model analysis, we arranged the model selection based on both the complexity of the terms and the variance of the random effects. When the maximal model could not converge or showed singularity, we first dropped the random correlation terms (once and for all, see the response to Comment #1b), and then dropped one random factor at a time, starting from the most complex interaction terms. When there were multiple terms with the same complexity, we compared the variances of the random effects in the last model and dropped the term with the least amount of variance. We repeated this selection process until we fitted the first model that converged and showed no singularity. We have revised our description of the model selection method in the manuscript accordingly (p22).

We have updated the information about the final model and the fixed effects of the model in the manuscript. We have also updated our model selection method in the OSF repository (link: <https://osf.io/6utkf/>). There you could also find two additional scripts that tracked the process of model selection.>>

b) The authors write that they first dropped random correlations, and then started dropping random slopes terms. However, most of the models (the main model for Exp 1b, 2a, and 2b) - both in the R code and as written in the paper text - contradict this stated strategy: Those models RETAIN (e.g.) the by-subjects random correlations, but DROP almost all of the by-subjects random effects. (But Exp 1a follows this stated strategy, so they differ between each other.) That is, this model did NOT first drop the by-subject random correlations and then start dropping random effects. It’s really important to both (1) have the description in the paper body match your actual model, and (2) to have a systematic and consistent strategy for pruning a model’s terms, as this can have a major effect on the model’s output.

<<Authors’ response: The reason that the exclusion of the random correlation was not symmetrical for the by-subject and the by-item random effects is because that we fitted models with and without random correlations in each iteration of the term dropping. We always started a model with random correlations. If that model did not converge, we would fit a model that dropped the random correlation for subjects and a model that dropped the random correlation for items. If both models converged, we selected the model based on the goodness-of-fit of the models. If one of the models converged, that model would be the final model. If neither of the models converged, we would fit a model with no random correlation. We did this iteratively each time after a term was dropped from the random model. Sorry we did not make it very clear in the manuscript.

In the newest version of the manuscript, we aligned the method of model selection with the description in the Results section of the manuscript. More specifically, now we started the data analysis with the maximal model, if that model could not converge, we fitted another model with the most complex random effect structure, but the random correlation was dropped. If the reduced model could not converge, we continued with the model selection strategy described in the response of comment #1a and never considered the random correlation again. We hope now the model selection method is consistent with our description in the manuscript. >>

c) In both the R code and the text body, only some of the factors appear to be tested. In both Exp 1a and 1b, there are models testing only these fixed effects:
-Prime Structure
-Trial Number
-Prime Structure x Head Noun
-Prime Structure x Task Difficulty
-Prime Structure x Head Noun x Task Difficulty
Where are the models testing the other fixed effects? Did you test, for example, for the main effect of Head Noun or Task Difficulty? If you didn’t test all factors, you should say that explicitly in the paper and justify why not. Even if some factors are not theoretically interesting, it is very non-standard to not even test for/report those factors if they were entered into your analysis.
The cross-experiment analysis of 1a vs. 1b similarly omits testing of a number of factors.
Strangely, in Exp 2a, the authors suddenly do test for the main effect of Problem Difficulty (but still not Head Noun), and Problem Difficulty x Lexical Overlap [sic].
But then testing for those two fixed effects disappears again in Exp 2b, which only tests for the 5 fixed effects I noted above.
So again we have a consistency problem.

<<Authors’ response: In the newest version of the manuscript, we only reported the fixed effects of the LME models (estimates, standard errors, and p-value) but not the results of model comparison. The tables of the fixed effects in the LME models for each experiment was moved from Appendix A to the results sections.

We further modified the report of results in such a way that we first reported theoretically interesting effects that were significant, followed by the theoretically interesting effects that were not significant, the unexpectedly significant effects, and ended with the other non-significant effects. This way, we hope the report of model results is more consistent.

Due to the model update and change in the reporting of the results, the three-way interaction between prime condition, head noun condition, and problem difficulty was no longer significant in Experiment 2a, while this interaction became significant in Experiment 2b. Accordingly, we deleted the section about the subset analysis in Experiment 2a and added a subset analysis in Experiment 2b (p43-p44). And the change of the patterns of the interactions led to some modification of the discussion about the lexical-specificity of the cognitive load effect on structure persistence (p53).>>

MINOR COMMENTS:
#2 Table 1: I understand, I had misunderstood what the numbers in Table 1 represented. The authors respond that they changed the title of the table to clarify this; I admit the title looks identical to the previous one except that one sentence was moved from the top to the bottom of the table, but I leave this to the authors to figure out.

<<Authors’ response: We made some further changes in order to clarify what each column represents in the table. First, in the first row of the header, we showed the name of the three independent variables. Second, we changed the wording of the table title to …*the proportion of s-genitive responses out of all s-genitive and of-genitive responses..*. Third, we added a column to the right of the table that reports the structure repetition effects in each head noun condition (i.e., the proportion of s-genitive in s-genitive condition minus that in of-genitive condition). >>

#3There are some drastic changes between different versions of lme4, so I suggest you report the version of the package that you used.

<<Authors’ response: We have reported the version of lme4 used in the analysis (p21).>>

#4 pg 29, line 292-293: The authors write "structural priming effect may persist over at least one intervening task, irrespective of what type of interference is employed." I do not think this is a fair statement; the authors have tested one type of secondary task (addition math problems); they don’t have evidence how any other type of task will or will not interfere.

<< Authors’ response: We have deleted this statement.>>

#5 The authors reference a condition called "Lexical Overlap" and "Overlap Condition" (pg. 36, line 439 and continuing on that page). Is this supposed to be “Head Noun Condition” or is this a new contrast they have introduced?

<<Authors’ response: We have unified the terms used in the manuscript>>

#6 The authors write (pg. 29, lines 281-283) that the "Inverse of Bayes factor" is 0.023 or 0.020. Do they really mean “inverse”? If that’s accurate, that means the Bayes factor is around 50 (1/0.020), which is very strong evidence in favor of H1 (cf Jeffreys 1961).

<< Authors’ response: Indeed, the inverse of Bayes factor (or BF_10_) here means the results that 1 is divided by the Bayes factor (BF_01_). It was argued that the bigger the inverse of Bayes factor is, the stronger the evidence that supports the alternative hypothesis (Raftery, 1995). That is why we followed the practice in previous literature and reported BF_10_ in the cross-experiment analyses. However, given we predicted that there would be no difference in the magnitudes of interactions between the two experiments, it is very much reasonable to only use Bayes factor (BF_01_) when we report the results of Bayesian analysis. So in the newest version of the manuscript, we decided to report Bayes factor (BF_01_) instead of its inverse (BF_10_).

We have made it clear that the Bayes factors that were reported in the manuscript were exclusively BF_01_. We then interpreted the BF_01_ only in terms of to what extent it supported or opposed the null hypothesis. This way, we hope to make the report of Bayes factors more consistent.>>

#7 The authors provide a short response to my previous question about whether the math problems provide enough of a cognitive load to disrupt processing in their Response letter. However, I didn’t see this in the manuscript itself. While I appreciate the personalized response, it would be useful if other readers could see this discussion as well.

<< Authors’ response: We have added parts in the manuscript that justified the design of the secondary task (p13, p35, and p47).>>

MINOR WORDING EDITS:
#8 pg. 29, line 291-292: “suggest that THE structural priming effect may persist”
#9 pg. 109, line 326-327: “One unexpected result in the priming experiments was a negative correlation between the critical trial number and the likelihood of s-genitive production.” --> “priming experiment” [singular], as you only found this negative correlation in Exp 1a, not 1b.

<<Author’s response: We have modified all the wording errors. Thank you for pointing them out.>>
